# Serum gonadal hormones levels and hypogonadism in ART naïve newly diagnosed HIV infected adult males in Mwanza, Tanzania

**DOI:** 10.1186/s12902-024-01581-w

**Published:** 2024-04-23

**Authors:** Shabani Iddi, Haruna Dika, Benson R. Kidenya, Samuel Kalluvya

**Affiliations:** 1 Department of Physiology, Weill Bugando School of Medicine, Catholic University of Health and Allied Sciences, Mwanza, P. O. Box 1464, Tanzania; 2 Department of Biochemistry, Weill Bugando School of Medicine, Catholic University of Health and Allied Sciences, Mwanza, P. O. Box 1464, Tanzania; 3 Department of Internal Medicine, Weill Bugando School of Medicine, Catholic University of Health and Allied Sciences, Mwanza, P. O. Box 1464, Tanzania

**Keywords:** Human immunodeficiency virus, Antiretroviral naïve males, Gonadal hormones, Hypogonadism

## Abstract

**Background:**

Human immunodeficiency virus/acquired immunodeficiency syndrome (HIV/AIDS) is an endemic chronic disease which is characterized with progressive depletion of CD4 T cells and increased susceptibility to opportunistic infections. Previous studies have associated HIV infection with increased hypogonadism. However, the prevalence of hypogonadism remained poorly defined and widely ranging in various studies. This study aims to evaluate the serum gonadal hormonal levels and hypogonadism in antiretroviral therapy (ART) naïve newly diagnosed HIV infected-males in Mwanza, Tanzania.

**Methods:**

This was a comparison study involving 81 ART naïve newly diagnosed HIV-infected adult males as study group and 81 apparently healthy HIV-negative males as comparison group. The participants in the study group and comparison group were matched by body mass index and age. Serum hormones [Total testosterone (TT), follicle stimulating hormone (FSH), luteinizing hormone (LH) and estradiol (E) were estimated. Serum testosterone < 300 ng/dl, or testosterone > 300 ng/dl with high LH and FSH (compensatory hypogonadism) were taken as markers of hypogonadism. Data were analyzed using STATA version 15.

**Results:**

The median serum testosterone level among ART naïve newly diagnosed HIV-infected adult males was significantly lower as compared to their comparison group (447 [259–534] versus 517 [396–605]; *p* = 0.0074) and shown to decrease with decreasing CD4 level. The median [IQR] serum FSH level among ART naïve newly diagnosed HIV-infected adult males was significantly higher than among their comparison group (3.8 [2.1–6.5] versus 2.6 [1.8–4.2]; *p* = 0.0086). The differences in serum LH and Estradiol were not statistically significant. Furthermore, the proportion of hypogonadism was significantly higher among ART naïve newly diagnosed HIV-infected adult males than in their comparison group (37.0% [30/81] versus 14.8% [12/81]; *p* = 0.0006). Out of these 30, 24 HIV-infected males had secondary hypogonadism, one had primary, and the remaining five had compensatory hypogonadism.

**Conclusion:**

Serum testosterone was lower and follicle stimulating hormone was higher among ART naïve HIV-infected males as compared to the HIV negative controls. Hypogonadism, mainly secondary, is common endocrine abnormality among ART naïve HIV-infected male patients in this study. HIV is associated with variations in gonadal hormones which may lead to sexual dysfunction in infected individuals.

## Introduction

Human immunodeficiency virus/acquired immunodeficiency syndrome (HIV/AIDS) is an endemic disease that contributes to a significant number of deaths among young adults worldwide [1]. According to the Joint United Nation Programme on HIV/AIDS (UNAIDS), there were 39 million people living with HIV worldwide in 2022 with Africa (WHO Africa regions) accounting for 69% of the global total [2]. In Tanzania, the number of people living with HIV has remained high with an estimated 1.7 million people living with HIV (PLWHA) as of year 2022. The UNAIDS report indicated that, between 2010 and 2022, new HIV infections have dropped by 38% and HIV– related deaths dropped by 51% [2]. However, the majority of new infections continue to occur in WHO Africa regions accounting for half of the global new HIV infections with more than three quarter of the cases occurring in Eastern and Southern Africa [2]. Therefore, more work is still needed to minimize the threat of HIV/AIDS.

Gonadal hormones are hormones produced by gonads (ovaries for females and testes for males), and include both steroids and peptide hormones. The major steroid hormones are estradiol and progesterone from the ovaries and testosterone from the testes, and are released under the influence of gonadotropins, luteinizing hormone (LH) and follicle stimulating hormone (FSH) from anterior pituitary gland. These hormones are very important for proper reproductive function [3]. Many alterations on endocrine functions including reproductive endocrine functions have been reported in association with HIV/AIDS in both early and late stages of HIV infection resulting in poor quality of life and significant morbidity and mortality [4]. These changes may be as a result of the opportunistic infections, direct effects of HIV, under-nutrition, infiltration by neoplasm, a complication of HIV treatment, or generation of cytokines [4–6]. For instance, studies done by Postel et al., 1994 [7] and Kaplan et al., 2000 [8] have demonstrated prevalence of hypogonadism of more than 70% among TB infected individuals, some of whom were HIV co-infected.

Hypogonadism is prevalent among people living with HIV/AIDS (PLWHA); commoner among men as compared to women [6]. Furthermore, HIV infected individuals have been reported to have low testosterone with raised serum LH and FSH levels [6, 9]. Studies have reported racial and/ethnic variations in sex/gonadal hormones [10–14] which may be due to genetic factors, socio-demographic factors, cultural and/ or environmental/dietary variations [12, 15, 16]. Most of the findings on the effect of HIV/AIDS on gonadal hormones documented were not observed in African setting such as Tanzania, and considering racial/ethnic variations in gonadal hormone levels, the extent of gonadal hormone decrease in HIV might differ in different populations, it is therefore necessary to assess the effect of HIV/AIDS on the serum gonadal hormone levels in HIV-infected males in Tanzania to determine whether there are differences in the gonadal hormone levels.

## Materials and methods

### Study design, setting and subjects

This was a comparison study designed to evaluate the serum gonadal hormonal levels and hypogonadism in newly diagnosed male subjects before starting ART in Mwanza, Tanzania. Study group individuals were the ART naïve newly diagnosed HIV-infected males and the comparison group were apparently healthy HIV negative male individuals matched for body mass index (BMI) and age. Matching of age and BMI was not strict and included by age ± 1 year and BMI ± 1 KgM^2^. Sample size was estimated using formula, for comparing two proportions (Kirkwood & Sterne, 2003) [17]. Using the value of the proportion of individuals with hypogonadism in the HIV-positive (25.9%) and control group (4.9%) in a study done in India [18], gave a minimum sample size of 51 individuals per group. A total of 81 individuals per group were recruited and used in this study.

All newly diagnosed HIV positive (diagnosed as per WHO guidelines 2015) males aged 18 years and above were recruited at Voluntary Counseling and Testing (VCT) centres at Sekou-Toure Regional Referral Hospital (STRRH), Bugando Medical Centre (BMC), Nyamagana District Hospital (NDH) and Ilemela District hospital (IDH) and included as study group. Patients with previous history of gonadal dysfunction, taking drugs known to affect the hormone levels (i.e. androgens, sex steroids, dehydroepiandrosterone, antiandrogens, anabolic agents, GnRH agonists and psycholeptic agents), having chronic liver disease, chronic kidney injury and chronic systemic illnesses such as tuberculosis (TB) and diabetes mellitus (DM) that might cause hypogonadism were excluded from the study. Comparison group was attending the VCT center at STRRH. A convenient sampling technique was used to include participants that met the inclusion criteria.

### Clinical assessment

All individuals were assessed clinically by detailed history taking and general physical examination including anthropometric measurements that included waist circumference (WC) and body mass index (BMI). Socio-demographic data including age, employment status, marital status and herbal medicine use status (whether used any herbal medicine within the past six months or not) were collected using a pre-structured questionnaire. Height was measured in the upright standing position using a stadiometer and. body weight was measured by a weighing scale, and BMI was then calculated by the formula: weight in kilograms divided by height in meters squared. WC was measured at the approximate midpoint between the lower margin of the last palpable rib and the top of the iliac crest using flexible plastic tape.

### Laboratory analysis

Five milliliters (mls) of blood sample were collected from each of the study participants between 8.00 and 11.00 AM and serum harvested. The serum was used for the estimation of Total Testosterone (TT) hormone, Follicle stimulating hormone (FSH), Luteinizing hormone (LH) and Estradiol (E) levels. The serum samples were stored at -20^o^C until analyzed.

The hormonal tests were done using chemiluminescence immunoassay (CLIA) techniques. The CLIA kits were obtained from the Snibe Co., Ltd, Shnzhen, China. The fully-auto chemiluminescence immunoassay analyzer model Maglumi 2000 (Snibe Diagnostic, China) was used to estimate serum hormones (TT, FSH, LH and E) according to the principles of CLIA and protocols given by the kit manufacturers. The CD4 + count was assessed by flow cytometry (Roche diagnostics).

### Criteria for definition of gonadal status

Hypogonadism was defined as a serum TT level of < 300ng/dl or a serum TT level of ≥ 300 ng/dl with high FSH (> 12 mlU/L) or LH (> 12 mlU/L) level [19]. Eugonadism was defined as normal TT and normal FSH and LH levels. Compensatory hypogonadism was defined as normal TT but high FSH or LH levels. Primary hypogonadism was defined as low TT levels with high FSH and LH while secondary hypogonadism was defined as low TT with low or normal FSH or LH [18, 19].

### Statistical analysis

A total of 81 study group participants and 81 comparable comparison group participants were enrolled in the study. The data obtained were analyzed using STATA software, version 15 (USA). We used Shapiro-Wilk normality test to assess the normality of continuous variables. Parametric continuous data were summarized as means with standard deviation (SD). Non-parametric continuous data were summarized as median with interquartile range (IQR). Outcome of interest were serum total testosterone (TT), follicle stimulating hormone (FSH), luteinizing hormone (LH) and estradiol (E) levels. To assess the statistical difference when comparing our outcome between the ART naïve newly diagnosed HIV infected adult male and non-HIV infected adult male as a comparison group, we used student’s t-test for the parametric outcome and Wilcoxon rank-sum test for non-parametric outcome. We used two sample proportion test to compare the significance of difference in proportion of hypogonadism between the ART naïve newly diagnosed HIV infected adult male and non-HIV infected adult male as a comparison group with the hypothesis that proportion of hypogonadism will be higher in ART naïve newly diagnosed HIV infected adult male than in their comparison group. To assess the significance of difference when comparing various patient characteristics (BMI, Waist circumference and CD4 cell count) between the ART naïve newly diagnosed HIV infected adult male with hypogonadism and HIV patients with no hypogonadism we used student’s t-test for the parametric characteristics and Wilcoxon rank-sum test for non-parametric parametric characteristics. In all analyses the difference was considered statistically significant at one-tailed p-value < 0.05.

### Ethical consideration

The study was approved by the Joint Catholic University of Health and Allied Sciences and Bugando Medical Center (CUHAS/BMC) research ethics and review committee with ethical clearance certificate number CREC/407/2019.The study participants provided written informed consent before enrollment into the study.

## Results

### Socio-demographic characteristics of study participants

A total of 81 ART naïve HIV infected adult males as study group and 81 non-HIV infected adult males as a comparison group matched by age and BMI were enrolled in the study. The mean age and BMI were 39.2 ± 10.5 years and 22.0 ± 2.5 Kg/M^2^ for the study group, and 38.9 ± 10.3 years and 22.2 ± 2.6 Kg/M^2^ for the comparison group respectively. More than half, 59.3% (48/81) of the HIV infected participants were not married while about three quarters, 72.8% (59/81) of the HIV negative control were married. The rate of herbal medicine use was higher among HIV negative males in the comparison group as compared to the HIV infected males (38/81 versus 18/81 respectively). Other characteristics are shown in Table 1.

**Table 1:** Socio-demographic characteristics of study participants

Variable	Study group Frequency (%)	Comparison group Frequency (%)
**Age (Years)**	39.2 ± 10.5*	38.9 ± 10.3*
**BMI (**Kg/M^2^**)**	22.0 ± 2.5*	22.2 ± 2.6*
**Waist Circumference (cm)**	78.9 ± 8.5*	85 0.9 ± 9.2*
**Marital Status**		
Married	10 (12.4)	59 (72.8)
Single	48 (59.3)	22 (27.2)
Divorced/separated	23 (28.4)	0 (00.0)
**Herbal Medicine use**		
Yes	18 (22.2)	39 (48.2)
No	63 (77.8)	42 (51.9)
**Employment status**		
Non self employed	19 (23.5)	11 (13.6)
Self Employed	54 (66.7)	67 (82.7)
Unemployed	8 (9.8)	3 (3.7)

*Mean ± SD

### Gonadal hormones

**Fig. 1:**
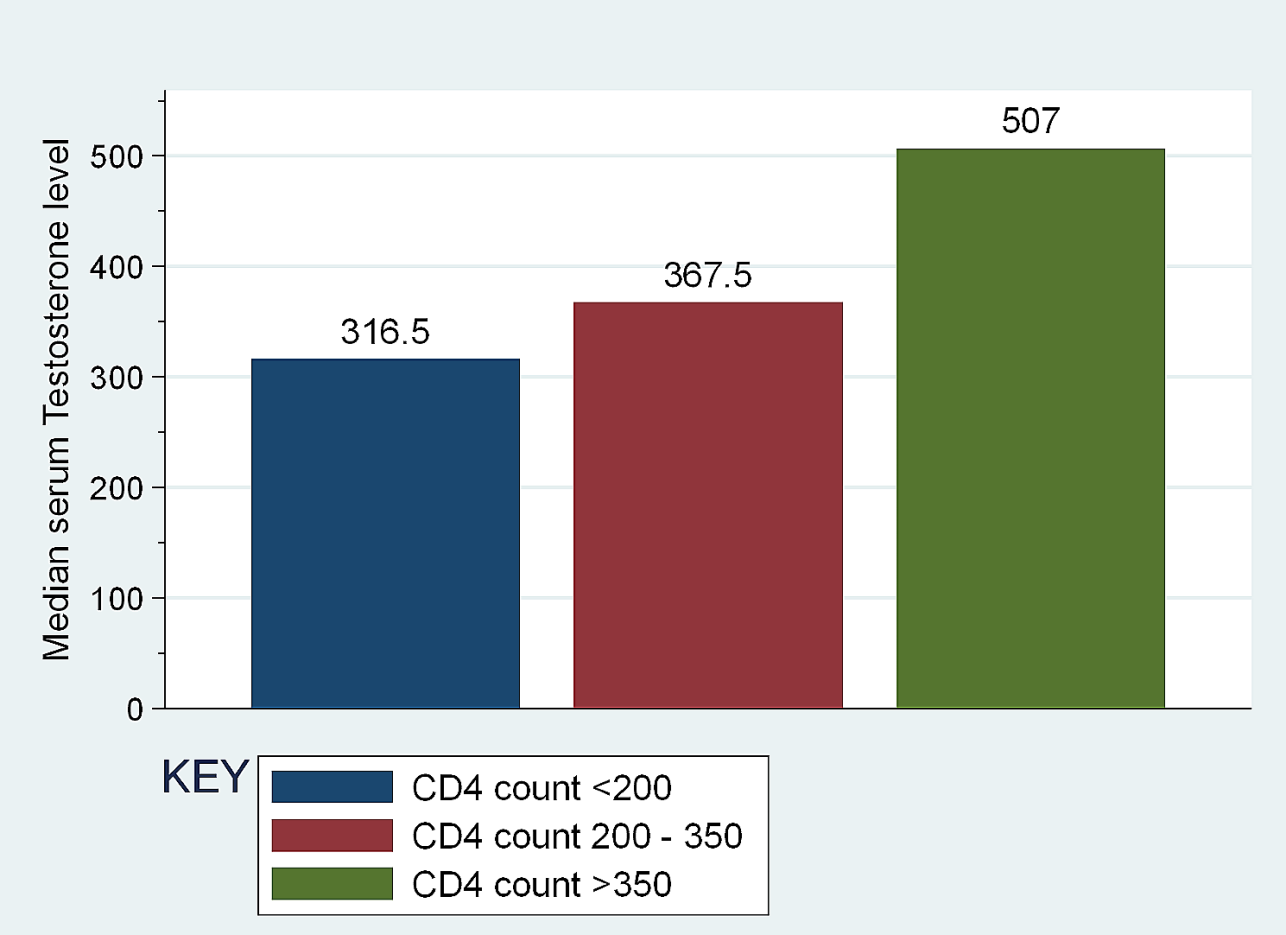
Median Testosterone level at different CD4 counts among the study group

The median [IQR] of serum TT level among study group, was significantly lower as compared to the comparison group (447 [259–534] versus 517 [396–605]; p *= 0.0074)* (Two-sample Wilcoxon rank-sum (Mann-Whitney) test) (Table 2). The median TT tended to decrease with decreasing CD4 count (Fig. 1). The median [IQR] of serum FSH level among study group showed significantly higher value as compared to comparison group (3.8 [2.1–6.5] versus 2.6 [1.8–4.2]; *p* = 0.0086) (Two-sample Wilcoxon rank-sum (Mann-Whitney) test). Serum LH and Estradiol levels did not significantly differ between the comparison groups (Table 2).

**Table 2:** Median serum hormonal levels in ART naïve newly diagnosed HIV infected males (Study group) and HIV negative comparison group

Variable	Study group Median [IQR]	Comparison group Median [IQR]	p-value
**Gonadal hormones**			
Total Testosterone (ng/dl)	447 [259–534]	517 [396–605]	0.0074*
FSH (mIU/L)	3.8 [2.1–6.5]	2.6 [1.8–4.2]	0.0086*
LH (mIU/L)	4.9 [3.9–6.4]	4.6 [3.7–5.9]	0.2867
Estradiol(pg/ml)	143.2 [108–237.4]	182.6 [140.2–232.5]	0.0626

*Significant

The proportion of hypogonadism was significantly higher among newly diagnosed HIV infected-drug naïve adult males than in their comparison group (37.0% [30/81] versus 14.8% [12/81]; *p* = 0.0006) (Two sample proportion test). Primary hypogonadism was seen in 1/81 (1.2%) of study group, secondary in 24/81(29.6%) study group and compensatory in 5/81 (6.2%) study group while in comparison group there were no primary hypogonadism but secondary and compensatory hypogonadism were seen in 11/81 (13.6%) and 1/81 (1.2%) respectively.

### Comparison of BMI, WC and CD4 level among study group with and without hypogonadism

There were significant lower BMI among study group (ART naïve newly diagnosed HIV infected males) with hypogonadism than among those without hypogonadism (21.3 ± 0.5 versus 22.4 ± 0.3) (Two-sample t test with equal variances; *p* = 0.0221). WC was higher among study group participants with hypogonadism than the study group without hypogonadism but the difference was not significant (Two-sample t test with equal variances; *p* = 0.0617). CD4 level was significantly lower among study group participants with hypogonadism than among study group without hypogonadism (Two-sample Wilcoxon rank-sum (Mann-Whitney) test; *p* = 0.0311). There were no significant difference in herbal medicine use status between the groups (Pearson’s χ^2^-test; p *= 0.461*) (Table 3).

**Table 3:** Mean/Median BMI, WC and CD4 level in ART naïve newly diagnosed HIV infected males with and without hypogonadism

HIV patient characteristics	Hypogonadism		Mean difference 95% [CI]	p-value
	Yes	No		
Mean BMI	21.3 ± 0.5	22.4 ± 0.3	1.14 [0.03–2.24]	0.0221
Mean WC	80.0 ± 1.1	77.0 ± 1.7	3.0 [-0.8–6.9]	0.0617
Median CD4	236 [IQR: 148–336]	353 [IQR: 231–401]	-	0.0311
Herbal medicine use n (%)			-	
Yes	8 (44.4)	10 (55.6)	-	0.461
No	22 (34.9)	41 (65.1)	-	

## Discussion

This study found a significant decrease in serum levels of testosterone in ART naive newly diagnosed HIV infected males compared with HIV negative comparison group. This decrease may indicate presence of hypogonadism among the studied population. This is in agreement with the findings of previous studies [20–22]. This decrease may be due to the direct effect of HIV on the gonads [23] or due to cytokine effect as HIV is known to upregulate tumor necrosis factor and interleukin 1 [24, 25], consequently leading to diminished testicular steroidogenesis.

There were significant higher median serum FSH levels, insignificant higher median LH levels among ART naïve newly diagnosed HIV infected males as compared with negative control. These differences might be due to the physiological connection between LH, FSH, Estradiol and testosterone. The secretion of testosterone from Leydig cells is regulated by LH and through negative feedback, testosterone reduces the levels of LH and FSH but too low testosterone allows increased secretion of LH and FSH. In a study by Yelwa et al. [20], the levels of FSH, LH and estrogen were found to be significantly higher among HIV positive and ART naïve males compared to negative control.

Circulating testosterone can undergo peripheral conversion to estrogen by action of Aromatase (estrogenic effect) [26]. Elevated serum LH and FSH concentrations on the testis have a stimulatory effect causing increased conversion of testosterone to estradiol [25]. Also earlier reports have shown occurrence of abnormal androgen metabolism which resulted in increased aromatization of testosterone to estradiol in HIV infected men [27]. A study by Ezeugwunne et al. [28]., confirmed this fact. In contrast to the previous study [28], the present study showed decreased estradiol level among ART naïve HIV infected males as compared with negative control but the difference was not statistically significance.

Hypogonadism is common among HIV infected males with prevalence between 29.0 and 89.7% without ART and 20–30% on ART [29, 30]. In this study hypogonadism was found to be significantly more common among HIV patients (37%) as compared to the normal population (14.8%). Secondary hypogonadism was the most common form of hypogonadism accounting for 80% HIV infected males. Similar to this study, a more recent meta-analysis study reported hypogonadism to be common in HIV patients as compared to control, but the prevalence of hypogonadism in the HIV was lower than that reported in a current study (overall 26%, and 33% & 19% when total testosterone alone and calculated free testosterone was considered respectively vs. 37%) [31]. The lower prevalence in the meta-analysis study may be contributed by most of the studies involving HIV patients who were already treated with HAART. Studies done in the pre-ART era reported higher prevalence of gonadal dysfunction caused by direct toxic effect by the virus on testicular tissue reducing quantity of Leydig cells [32].The current study shows secondary gonadal dysfunction to be higher than primary. This observation is similar to the findings of the other studies done after introduction of more effective HIV therapy [14, 18, 21]. Various mechanisms have been suggested to explain the effect on the hypothalamic-pituitary-gonadal (HPG) axis which include poor health status, undernutrition, frailty, opportunistic infections, antiviral drugs, the virus itself and increased visceral fat [33]. Also insulin resistance in lipodystrophy which leads to hyperglycemia was found to be associated with low testosterone levels in various studies indicating that insulin resistance may cause hypogonadism [34]. Interestingly the finding of this study compared to previous studies showed presence of hypogonadism and/or reduced testosterone in naïve patients not exposed to ART, and therefore it means that the virus itself or comorbid condition are able to influence the hypothalamic-pituitary-gonadal axis. However, in this study we excluded individuals with chronic illnesses including TB and Diabetes mellitus.

There were significant lower BMI and insignificant higher WC among HIV patients with hypogonadism as compared with HIV patients without hypogonadism. This finding supports the observations made by other scholars. For instance, Rietschel et al. [35], found hypogonadism to be more common among patients with AIDS wasting, but no correlation was found with CD4 count. Gomes et al. [21], showed 18.1% of patients with testosterone deficiency presented with visceral obesity (as measured by waist circumference > 102 cm), contrasting to 7.5% of eugonadal individuals. The effect of gonadal hormone levels on the development of lipodystrophy is not largely known [34, 36]. Testosterone is one of the determinants of regional distribution of fat and body composition, whereby its deficiency is associated with accumulation of visceral fat. The other mechanisms which favor accumulation of visceral fat include an increase in tissue sensitivity to glucocorticoids, reduction of adipokines, and a reduction of peroxisome proliferator-activated receptor (PPAR-γ) activity [21, 37]. Various studies have shown low levels of testosterone correlate not only with central fat accumulation but also with higher insulin levels which suggest testosterone also has an effect in insulin sensitivity in HIV-lipodystrophy [34].

The findings also show a significant decrease in the levels of CD4 count among HIV patients with hypogonadism as compared with those without hypogonadism This is similar to the finding of the previous study [18]. HIV infection is characterized with progressive depletion of CD4 count and has been reported to be associated with changes in serum gonadal hormones levels [6, 18, 38, 39]. Studies by Bajaj et al. [18], and Meena et al. [6], found a direct correlation between serum testosterone and lower CD4 counts among HIV patients.

Hypogonadism in HIV patients is mainly due to secondary hypogonadism in this study. Previous studies have shown poor health status associated with a worse gonadal function in HIV patients [39, 40]. Similarly, the current study showed significantly reduced CD4 count that represents a marker of advanced HIV infection and poor health status, in HIV patients with hypogonadism than HIV patients without hypogonadism. Studies have shown a link between poor health status, frailty, total body fat and visceral adipose tissue in HIV patients [41, 42]. Recently a study by De Vicentis et al. showed low serum total testosterone (TT) and calculated free testosterone (cFT) to be independently associated with multi-morbidity and frailty in young and middle aged HIV infected men [39]. Further the same study found frailty to be inversely related to TT especially cFT, while it was directly related to BMI and body fat, particularly its visceral component among others [39]. This shows existence of a link between total body fat, especially visceral fat and frailty, therefore the relation between sex steroids and overall health status. This leads to the hypothesis that increased body fat and in particular visceral fat may boost TT decrease and that total body fat is linked to and/ or is a biomarker of poor health status [39]. The boost in TT decrease may probably be through an increased aromatization [43] or other mechanism such as inhibition of gonadotropin secretion through leptin resistance or adipokine release [44, 45].

In our study there were significant lower BMI and insignificant higher WC among HIV patients with hypogonadism as compared with HIV patients without hypogonadism. The higher WC (which correlates positively with visceral fat) among HIV patients with hypogonadism though not significant is in line with the finding of previous studies which indicated low testosterone level correlates with central fat accumulation [34]. This indicates the more secondary hypogonadism observed in these patients could be due to the boost of TT decrease by visceral fat by the mechanism as discussed above.

Traditional herbal medicine and complementary alternative medicine are commonly used by ART naïve HIV patients [46, 47] and also the use is on the rise among the general population for treatment of various conditions including sexual dysfunction [48–53]. However, some herbal medicines are known to have antifertility properties by various mechanisms including inhibiting 5-alpha reductase (a factor that converts testosterone into dihydrotestosterone), reducing gonadotropins and testosterone secretion, increasing the testosterone affinity for sex specific proteins among others [54] hence could be one of the contributing factors to hypogonadism. This study found higher herbal medicine use in the HIV negative control as compared to HIV-infected counterparts. The higher rate of herbal medicine could be due to the increased dependence on traditional medicine for primary health care of up to 80% of the population in Tanzania [55]. We did not find a significant difference in herbal medicine use between HIV patients with and without hypogonadism.

Limitation of our study include lack of the SHBG measurement and, therefore calculated free serum testosterone which may have underestimated the prevalence of hypogonadism in HIV patients since measurement of SHBG has been highly recommended in men with HIV in addition to serum LH and TT due to the possible rise in serum SHBG in these patients [56–58]. Another limitation is that, testosterone levels were determined using an immune- assay technique, whereas mass-spectroscopy is often considered “gold-standard” but is not commonly used because it is expensive and not widely available. However, the immuno-chemiluminescence assay used in the determination of the gonadal hormone values is internationally certified and widely used in clinical practice to diagnose and guide treatment in patients with gonadal dysfunction. Further the study is limited by failure to rule out some disease conditions which may also cause hypogonadism such as Cytomegalovirus (CMV), Mycobacterium avium complex (MAC), Cryptococcus neoformans infections and infiltrating neoplasms like Kaposi’s sarcoma,

## Conclusion

This study revealed lower serum testosterone (TT) and higher follicle stimulating hormone (FSH) among ART naïve HIV males as compared to HIV negative comparison group. It is further concluded that hypogonadism is a common endocrine abnormality among HIV– infected male patients affecting one out of three HIV males. Secondary hypogonadism was more common than primary hypogonadism among HIV infected males. HIV is associated with variations in gonadal hormones which may lead to sexual dysfunction in infected individuals.

## Data Availability

The datasets used and/or analysed during the current study are available from the corresponding author on reasonable request.

## Abbreviations

AIDS: Acquired immunodeficiency syndrome
ART: Antiretroviral therapy
E: Estradiol
FSH: Follicle stimulating hormone
HIV: Human immunodeficiency virus
LH: Luteinizing hormone
PLWHA: People living with HIV/AIDS
TT: Total testosterone
UNAIDS: United Nations program on HIV/AIDS
